# Socio-geographical factors and vulnerability to leptospirosis in South Brazil

**DOI:** 10.1186/s12889-023-16094-9

**Published:** 2023-07-07

**Authors:** Alessandra Jacomelli Teles, Bianca Conrad Bohm, Suellen Caroline M. Silva, Fábio Raphael P. Bruhn

**Affiliations:** 1 Municipal Health Department of Herval, Rio Grande Do Sul, Brazil; 2 Postgraduate Program in Veterinary, Federal University of Pelotas, Capão Do Leão, Rio Grande Do Sul, Brazil; 3 Department of Preventive Veterinary Medicine, Federal University of Pelotas, Capão Do Leão, Rio Grande Do Sul, Brazil

**Keywords:** Health vulnerability, Epidemiology, SINAN, Public health surveillance

## Abstract

**Background:**

Leptospirosis, caused by the *Leptospira* bacteria, is an acute infectious disease that is mainly transmitted by exposure to contaminated soil or water, thereby presenting a wide range of subsequent clinical conditions. This study aimed to assess the distribution of cases and deaths from leptospirosis and its association with social vulnerability in the state of Rio Grande do Sul, Brazil, between 2010 and 2019.

**Methods:**

The lethality rates and incidence of leptospirosis and their association with gender, age, education, and skin color were analyzed using chi-square tests. The spatial relationship between the environmental determinants, social vulnerability, and the incidence rate of leptospirosis in the different municipalities of Rio Grande do Sul was analyzed through spatial regression analysis.

**Results:**

During the study period, a total of 4,760 cases of leptospirosis, along with 238 deaths, were confirmed. The mean incidence rate was 4.06 cases/100,000 inhabitants, while the mean fatality rate was 5%. Although the entire population was susceptible, white-colored individuals, males, people of the working-age group, along with less-educated individuals, were more affected by the disease. Lethality was higher in people with dark skin, and the prime risk factor associated with death was the direct contact of the patients with rodents, sewage, and garbage. The social vulnerability was positively associated with the incidence of leptospirosis in the Rio Grande do Sul, especially in municipalities located in the center of the state.

**Conclusions:**

It is evident that the incidence of the disease is significantly related to the vulnerability of the population. The use of the health vulnerability index showed great relevance in the evaluation of leptospirosis cases and can be used further as a tool to help municipalities identify disease-prone areas for intervention and resource allocation.

## Background

Leptospirosis, caused by the spirochete of the genus *Leptospira* sp., is an emerging zoonotic disease worldwide. Till now, more than 22 *Leptospira* species have been identified worldwide, harboring over 300 serovars [1]. The disease is a zoonosis that affects humans and several species of wild, synanthropic, and domestic animals, including carnivores, rodents, primates, and marsupials, which can become reservoirs and facilitate further spread in the environment [1, 2].

Leptospirosis is a major public health problem in Brazil, with potential social, sanitary, and economic impacts that can be attributed to its transmission capacity in vulnerable populations, resulting in higher hospitalization costs, loss of working days, and a higher lethality rate in Brazil [2, 3]. The disease has a high incidence in the country, with an annual average of 3,926 confirmed cases and a lethality rate of 8.9%, between 2007 and 2016, with the highest number of cases registered in the Southeast and South regions of the country [4]. In 2010, Rio Grande do Sul reported an incidence of approximately 5 cases per 100,000 inhabitants, which was significantly greater than the national average of 1.9 cases per 100,000 inhabitants [5].

The occurrence of leptospirosis and others neglected tropical diseases is determined by complex interactions between hosts, climate, transport networks, population density, and nonplanned urbanization, which lead to inadequate infrastructures, social inequalities and access to health, lack of sanitation, and deforestation, among others [6, 7]. Therefore, the outbreak of epidemics is more common in susceptible populations that have been affected by natural disasters like floods or encounter inadequate basic sanitation needs, as well as are vulnerable to rodent infestation. Several epidemiological investigations based on the interactions between the agent and the susceptible host indicate that the home, as well as the workplace environment, are the probable sites of infection that mostly affects people in the working-age population aged 20–49 years [8, 9].

Leptospirosis is a neglected tropical disease, hence, reliable data on the incidence and prevalence of the disease in regions with different environmental, demographic, and infrastructural characteristics are still limited [6, 9]. Therefore, we consider that the analysis of real data on the incidence of confirmed leptospirosis cases collected from information systems derived from the official surveillance of the disease in Brazil could contribute to understand its dynamics of occurrence and elucidate the difficulties in disease control. Furthermore, since Leptospirosis has a high incidence in southern Brazil, a prompt risk assessment will help health care staff in developing specific control policies as well as providing primary care services to vulnerable populations.

Socioeconomic and environmental determinants could influence the occurrence of epidemics in an unequal way, especially in Brazil, where there are huge socioeconomic disparities including access to public health and basic sanitation [10, 11]. This study therefore aims to investigate the incidence and lethality of leptospirosis in the Rio Grande do Sul and describe the relationship between incidence, lethality and other socio-environmental vulnerability indicators within the state. These findings are expected to support health care providers in developing more effective strategies for controlling leptospirosis in higher incidence regions like southern Brazil.

## Methods

### Study design

This study is a retrospective ecological study using aggregated data collected during the routine surveillance of leptospirosis by Ministry of health of Brazil in the Rio Grande do Sul between 2010 and 2019. In brief, we analyzed the spatial distribution of leptospirosis incidence and determined the relationship between incidence, lethality, and various social determinants (age, gender, education, and skin color). Furthermore, we evaluated using spatial regression analysis, the association between leptospirosis incidence and indicators of environmental and social vulnerability.

### Study area

**Fig. 1:**
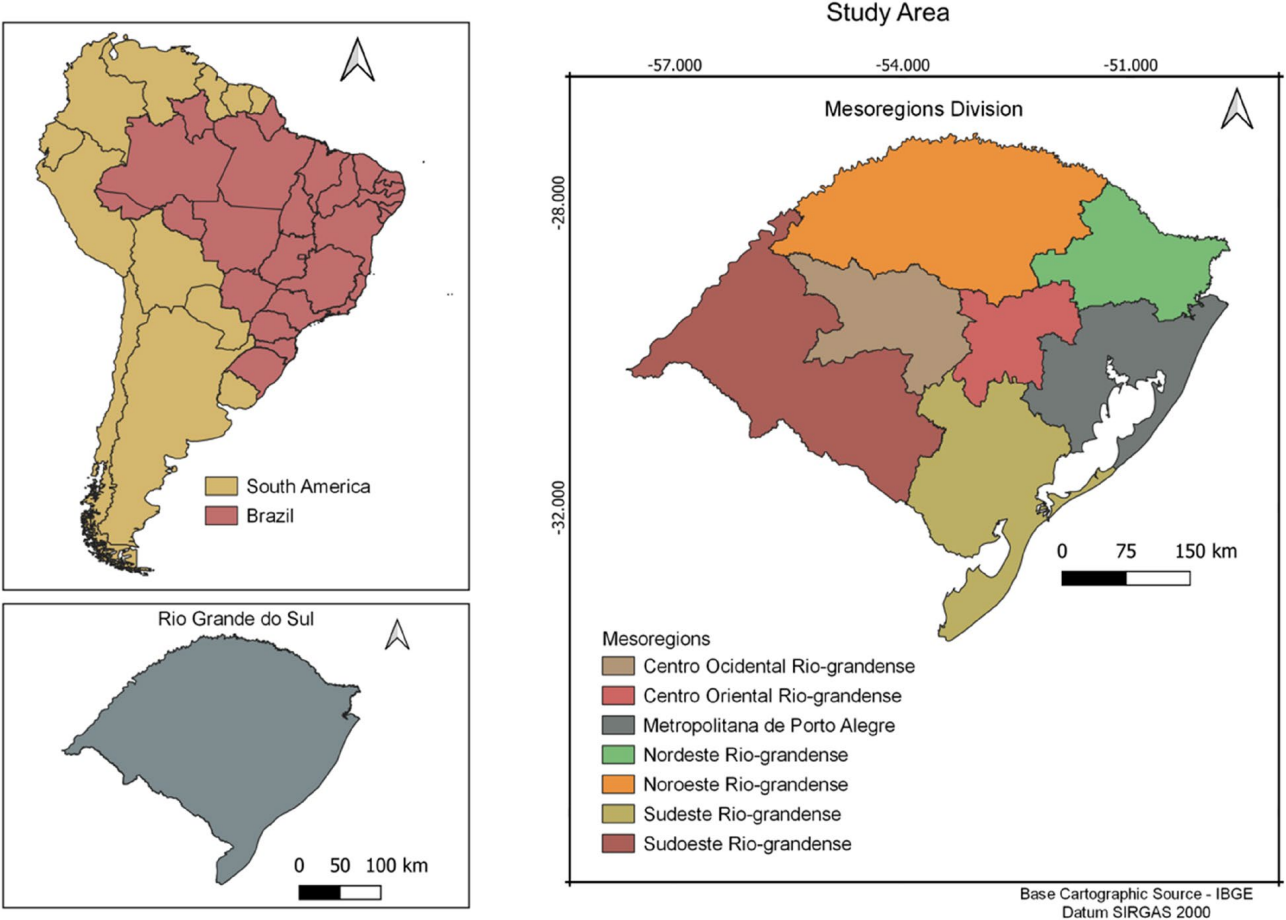
Location of the Rio Grande do Sul state in Brazil, with its divisions by mesoregions (Source; Shape file from IBGE, 2019, done using Qgis version 3.28). https://www.ibge.gov.br/geociencias/downloads-geociencias.html

Rio Grande do Sul is the southernmost state in Brazil and is divided into 497 municipalities, with an estimated population of 11,422,973 inhabitants, as well as an area of 281,730.223 km^2^in 2020 (Fig. 1). Although the state has a population density of 37.96 inhabitants/km^2^, 85.1% of the population lives in urban areas with a Human Development Index of 0.746 [12]. Its geographical position lies between the parallels 27°03” 42” and 33°45 “09” of South latitude and 49°42 “41″ and 57°40 “57″ of West longitude. The climate is subtropical, with seasonal temperature variations, like hot summers and harsh winters. Although the average temperature varies between 15 °C to 18 °C, the rainfall volume is different between the diverse regions, with an average annual precipitation of 1,500 mm [13].

The literacy rate is above 95%. According to statistical data from 2017, the Gross Domestic Product (GDP) *per capita* is close to R$37 thousand/year. In 2015, 93.7% of the population had access to direct or indirect garbage collection, whereas 40% had sewage collection networks around their homes [12].

There are three major economic regions: 1) the southern region, which contains a greater proportion of land, large livestock farms as well as mechanized planting of rice, soybeans, and wheat but faces higher income inequality; 2) the northeast region, which includes the state capital, and has more industries and predominantly small properties; and 3) the northern region, mostly colonized by European immigrants, and has a higher forest coverage, valleys and plains with small agricultural lands [13] (Fig. 1).

### Data collection

Aggregated leptospirosis case data were obtained from the Notifiable Diseases Information System database (SINAN)^1^of the Ministry of Health [14, 15]. Patient data related to age, gender, education, skin color, death, and the probable sites of infection in RS were analyzed. The study cases were confirmed by clinical-epidemiological or laboratory criteria in accordance with the Epidemiological Surveillance Guide of the Ministry of Health [16].

For laboratory confirmation, the suspected must present a reactive ELISA-IgM result, associated with seroconversion by MAT, that is, a negative result in a first collection (acute phase) followed by a positive result (with titer > 200) in a second sample collected 14 days after the first one [16]. In addition, those patients with a fourfold increase in the MAT titer between the first and second samples are also considered laboratory confirmed cases, or when the suspect has a titer greater than 800 in the MAT, even in just one blood sample [16].

Regarding the clinical-epidemiological confirmation, a suspected patient is considered confirmed when he has fever and clinical alterations in liver, kidney, or vascular functions, associated with epidemiological history (contact with floods, sewage, garbage, with a confirmed case or residence in an area of risk for leptospirosis) and, for some reason, has not collected material for specific laboratory test or presented a negative result (ELISA-IgM or MAT) in a single blood sample collected before 7 days of the onset of symptoms [16].

All confirmed cases of leptospirosis in Rio Grande do Sul that were notified to SINAN between 2010 and 2019 were included, without applying any inclusion criteria.

### Statistical analysis

The morbidity and lethality of the disease in RS were characterized through two dependent variables: (i) incidence rate (IR) and (ii) lethality rate (LR).

- (i) IR = (leptospirosis cases/population) × 100,000
- (ii) LR = (deaths from leptospirosis/cases of leptospirosis) × 100

Bivariate statistical analysis was carried out for the social determinants: age, gender, education, and skin color, as well as the deaths due to leptospirosis using the chi-square (χ2) or Fisher’s exact tests whenever necessary. The variables used in these analyzes were the same as those available on SINAN. Age categorization was performed at the intervals of 5 and 10 years, following the standardization method used in the 2010 IBGE census [17]. For the variables associated with a minimum confidence level of 95% (*p* < 0.05), the relative risk (RR) and its 95% confidence interval (IC.95%) were calculated by chi-square or Fisher’s exact tests. IBM SPSS Statistics 20.0 software was used for these analyses, considering a minimum confidence level of 95% (*p* < 0.05).

### Spatial modeling

The spatial distribution of leptospirosis was calculated by using the terrestrial geographic coordinate system containing ordered pairs of coordinates (x,y). Thus, the association between the incidence rate of leptospirosis and social vulnerability was evaluated and represented as the health vulnerability index (HVI). HVI was collected from the IPEA database of each municipality in the Rio Grande do Sul and correlated with the HVI data obtained in 2010.

The HVI is an index that helps in identifying hidden social exclusions and vulnerability in the Brazilian population for a deeper understanding of poverty, which is mostly presumed as a lack of sufficient monetary resources. This index was composed of sixteen indicators encompassing three dimensions: urban infrastructure, human capital, as well as income, and work, thereby allowing precise mapping of exclusion and social vulnerability for Brazilian municipalities (according to the municipal grid of the 2010 Demographic Census). In HVI, the score ranges from 0 to 1; the higher the score, the greater the social vulnerability of a municipality [18].

In this study, the HVI was evaluated as a predictor of the incidence rate of leptospirosis in each municipality of Rio Grande do Sul over the years through Ordinary Least Square (OLS) and Geographically Weighted Regression (GWR) linear regression analyses. Initially, an OLS regression model was built to obtain the global regression equation. After verifying the statistically significant association and the regression prerequisites, thematic maps of the GWR regression were constructed to evaluate the differences in the relationships between the incidence rates of leptospirosis in the different cities of the state.

OLS is a traditional regression method that estimates the global regression coefficient, which is constant over space (Eq. 1).

1 $$y={\beta }_{0}+{\Sigma }^{P}\beta ixi + \varepsilon$$

In this equation, y is the dependent variable, β_0_ is the intercept, X_i_ is the independent variable, β_i_ is the regression coefficient, ε is the error term, and p is the number of independent variables.

The OLS regression analysis was carried out considering stationarity assumption sand normality in the distribution of errors utilizing studentized Bruesch-Pagan and the Jarque–Bera tests, respectively. In addition, the representativeness of the independent variables over the dependent variables was estimated by calculating the adjusted R^2^ for each constructed model; R^2^ presents the predictive capability of a regression model to fit the measured values of the dependent variables [19]. Furthermore, global Moran’s I statistics were applied to verify whether the assumption of the regression models that the residuals show a random spatial distribution is in accordance with our OLS and GWR results. The global Moran’s I statistics are a measure of spatial autocorrelation and have been widely used to confirm the adequacy of regression models [20, 21].

GWR is an extension of OLS regression that allows local variation in parameters for exploring spatial non-stationarity in a sample^21^. In Eq. (2), the sample location (u, v) is added to the regression equation for estimating local parameters.

2 $$y_i=\beta_0(u_i,\;v_i)+\sum{\mathrm\beta}_{\mathrm k}(u_i,\;v_i)x_{\mathrm{ik}}+{\mathrm\varepsilon}_{\mathrm i}$$

In this equation, y_i_ is the dependent variable for location i, u_i,_ e, and v_i_are the coordinates of location i, β_0_(u_i_, v_i_) is the intercept at location i, β_k_ (u_i_, v_i_) is the estimate of the local parameter for an independent variable x_ik_ in location i, and ε_i_ is the error term.

After selecting the significant independent variables by OLS regression, GWR regression models were built to evaluate the differences in the relationships between the indices indicators, and the incidence rate of leptospirosis in different municipalities of Rio Grande do Sul. Thus, thematic maps of the coefficient and R^2^ were constructed for each tested variable, in addition to the error map, to verify the randomness in the distribution of the models’ error between the municipal coverage areas. The cross-validation Bandwidth method was used to select the number of neighbors to be considered in estimating the regionalized regression equations [22]. All spatial statistical analyzes were performed using GeoDa1.8.10 (Spatial Data Science Center, University of Chicago, Chicago, Illinois, IL, USA) and ArcGis 10.3(Open Source Geospatial Foundation, Beaverton, Oregon, OR, USA) software, respectively.

The study was approved by the Ethics Committee of the Faculty of Medicine of the Federal University of Pelotas, CAAE 46714421.0.0000, in accordance with all ethical principles and current legislation for research involving human beings. In this way, the patient data was kept confidential and was used only for research purposes.

## Results

From 2010 to 2019, a total of 4,760 confirmed cases of leptospirosis were reported in the Rio Grande do Sul, along with 238 (5.4%) deaths in the study period (mean IR/period = 4.06/100,000 inhabitants and mean LR/period = 5%). Additionally, 91.1% (4,334) and 7.2% (344) of the total cases were selected based on the clinical-laboratory and clinical-epidemiological criteria, respectively.

**Fig. 2:**
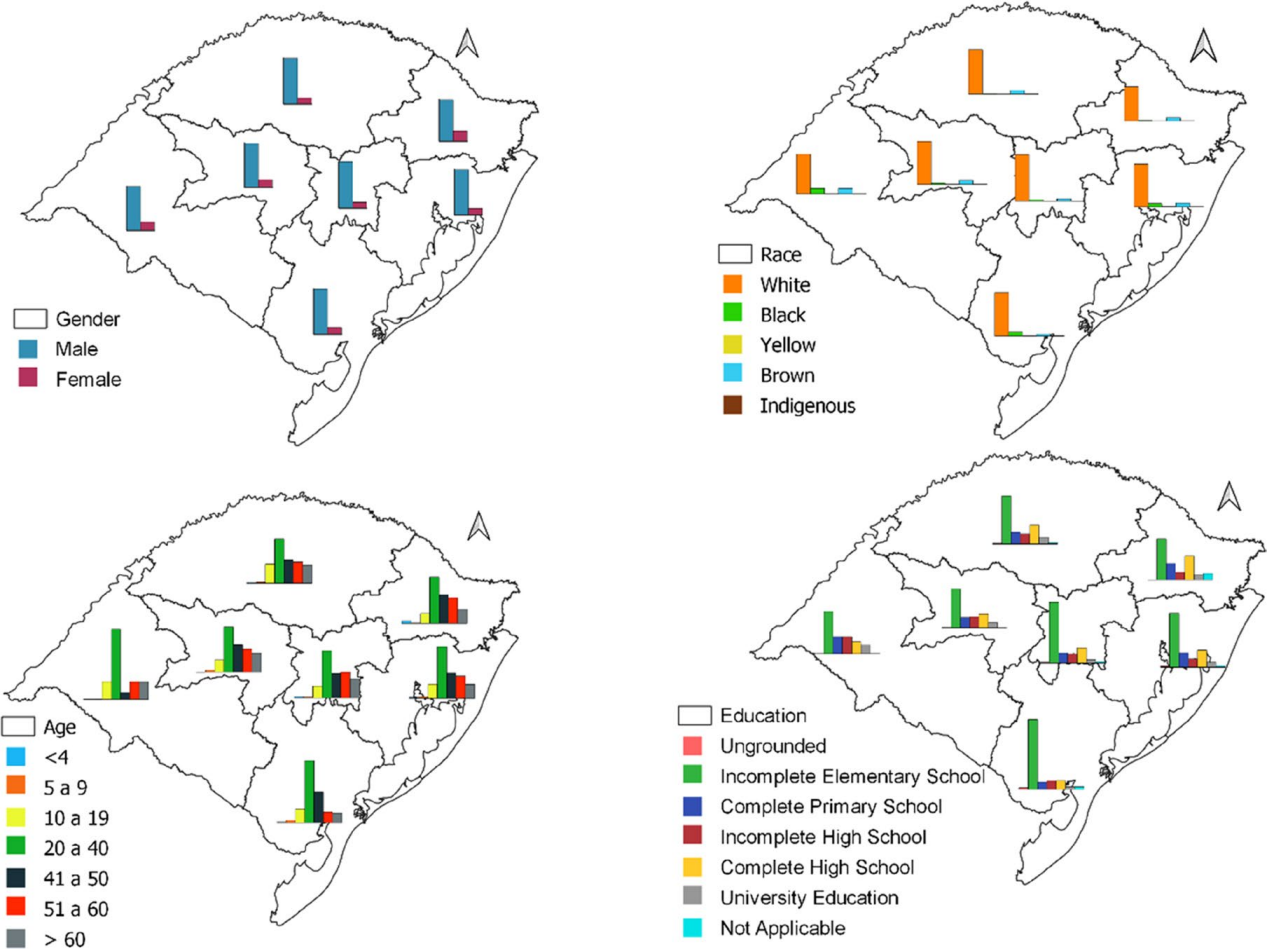
Spatial distribution of leptospirosis according to gender, skin color, age, and education in different mesoregions of Rio Grande do Sul, Brazil, 2010 and 2019 (Source; Shape file from IBGE, 2019, done using Qgis version 3.28). https://www.ibge.gov.br/geociencias/downloads-geociencias.html

The incidence of leptospirosis was significantly higher in men (87.1%; 4,145), with self-appointed white skin color individuals (88.6%; 3,995), and in the age group between 20 and 50 years (58.2%; 2,698), with incomplete primary education (56.3%; 1,636) (Table 1). Leptospirosis cases according to gender, race, age, and education in different mesoregions of Rio Grande do Sul are shown in Fig. 2.

**Table 1:** Demographic variables of patients with confirmed cases of leptospirosis in Rio Grande do Sul, Brazil, 2010 to 2019

Variable	N %
**Gender**	
Male	4145 (87,1%)
Female	615 (12,9%)
**Race**	
White	3995 (83,9%)
Black	207 (4,3%)
Yellow	15 (0,3%)
Brown	292 (6,1%)
Indigenous	14 (0,3%)
Missing Data	237 (5%)
**Age Group**	
0 a 4	32 (0,7%)
5 a 9	48 (1%)
10 a 19	515 (10,8%)
20 a 40	1829 (38,4%)
41 a 50	932 (19,6%)
51 a 60	817 (17,2%)
> 60	587 (12,3%)
**Education**	
Ungrounded	31 (0,7%)
Incomplete Elementary School	1674 (35,2%)
Complete primary education	372 (7,8%)
Incomplete high school	281 (5,9%)
Complete high school	477 (10,0%)
University education	150 (3,2%)
Not applicable	50 (1,1%)
Missing Data	1249 (36,2%)
**Area of infection**	
Urban area	1574 (33,1%)
Rural area	2157 (45,3%)
Periurban	160 (3,4%)
Missing Data	869 (18,3%)
**Infection ambience**	
Dosmetic	1665 (34,6%)
Work	1335 (27,4%)
Leisure	482 (10,1%)
Other	205 (4,3%)
Missing Data	1126 (23,6%)
**Work Disease**	
Yes	1528 (32,1%)
No	2298 (48,3%)
Missing Data	934 (19,7%)

The occurrence of work-related leptospirosis cases was 40% (1,528 cases), although, in 19.6% of the cases, this piece of information was not present. Considering the occupational variability, 37.5% (1,035) of the cases were related to agriculture and fishing. Additionally, most of the cases (2,157; 45.3%) were linked to rural areas, 1,665 cases (35%) were associated with the home environment, followed by workplace transmission (28%) and leisure activity areas (10%).

Our results show that it was found that IR was higher in adults aged 51 to 60 years, followed by people aged 41 to 50 years, in both the genders (Table 2). Overall, there were more cases of leptospirosis in males (87.1%); the incidence in men (7.76 cases/100,000 inhabitants) was seven times higher than in women (1.08 cases/100,000 inhabitants). The LR increased between men and women over 60 years; however, it was observed that the LR was significantly higher in males. Regardless of gender, it was found that older people have a higher risk of dying from the disease.

**Table 2:** Incidence rate and lethality rate of leptospirosis according to age group and gender in the Rio Grande do Sul, Brazil,2010 to 2019

Age group	Cases		Deaths		Incidence rate (cases / 100,000 100inhabitants)^a^		Lethality rate (%)	
Years	F	M	F	M	F	M	F	M
0–4	4	18	0	0	1.26	5.49	0.0	0.0
5 – 9	18	35	0	0	5.07	9.49	0.0	0.0
10 – 14	31	161	0	5	7.33	36.71	0.0	3.11
15 – 19	36	275	0	5	8.31	62.16	0.0	1.82
20 – 30	91	813	3	12	10.32	92.05	3.30	1.48
31–40	94	790	1	22	11.92	103.28	1.06	2.78
41 – 50	111	799	6	45	14.03	107.70	5.41	5.63
51 – 60	106	684	7	50	15.87	112.14	6.60	7.31
> 60	102	468	11	65	12.26	74.59	10.78	13.89
Total	593	4,043	28	204	10.80	77.67	4.72	5.05

^a^Population estimate according to data from the IBGE 2010 census, *F* female, *M* male

Individuals with self-appointed white skin color accounted for the largest number of cases of leptospirosis (88.3%), followed by dark (6.5%), black (4.6%), yellow (3%) skin colors, and indigenous (3%) populations, while the lethality was higher in brown skin colors individuals (8.2 cases/100,000 inhabitants), followed by indigenous (7.1 cases/100,000 inhabitants), black (6.7 cases/100,000 inhabitants), yellow (6.6 cases/100,000 inhabitants) and white (4.5 cases/100,000 inhabitants) skin color individuals. Furthermore, it was observed that the risk of death from leptospirosis in the Rio Grande do Sul is almost twice as high in black skin color individuals as compared to white skin color individuals (*p* = 0.005; RR = 1.898; 95% CI = 1.218 – 2.958). Regarding the patients' education levels, in 1,725 (36.2%) of the cases this information was missing. A greater number of cases of leptospirosis was observed in people who had incomplete elementary school education (*n* = 1,674; 56%), followed by people with complete high school (*n* = 477; 16%). It was found that people with a lower level of education had a higher risk of death from the disease (*p* < 0.05, Table 3).

**Table 3:** Association between schooling and deaths from leptospirosis in the Rio Grande do Sul, Brazil, 2010 to 2019

Schooling	Cases^a^	Deaths	*p*-value^b^	Relative risk (95% CI)
Illiterate	31	5	-	Ref
Incomplete elementary school	1,674	69	0.009	0.224 (0.083 – 0.600)
Complete elementary school	372	11	0.005	0.158 (0.051 – 0.490)
Incomplete high school	281	5	0.001	0.094 (0.026 – 0.347)
Complete high school	477	10	0.001	0.111 (0.035 – 0.350)
Incomplete higher education	66	0	0.003	0.839 (0.719 – 0.979)
Complete higher education	84	0	0.001	0.839 (0.719 – 0.979)
Incomplete elementary school	1,674	69	-	Ref
Complete elementary school	372	11	0.185	-
Incomplete high school	281	5	0.033	0.421 (0,168 – 1,054)
Complete high school	477	10	0.022	0.498 (0.255 – 0.975)
Incomplete higher education	66	0	0.066	-
Complete higher education	84	0	0.032	0.959 (0.949 – 0.968)

*Ref.* reference category, *CI* confidence interval

^a^in 1,725 (36.2%) of the cases the information about schooling was missing in the information system

^b^Chi-square test; only the reference categories that showed statistically significant associations (*p* < 0.05) with relation to other categories

Regarding the possible risk factors, it was observed that the factors which were significantly associated with death from leptospirosis were the patient’s previous contact with cesspools or sewers, streams, or lakes, directly with rodents, the farming environment, inadequate grain storage, and garbage and debris removal (*p* < 0.05).On the other hand, risk factors like contact with floods, location with signs of rodents, humans, and infected animals, were not associated with death from the disease in the Rio Grande do Sul (*p* > 0.05, Table 4). While evaluating the direct contact of confirmed cases with infected humans and animals, it was observed that 28.4% and 30.8% of the cases did not produce any information regarding this variable.

**Table 4:** Association between risk factors and death from leptospirosis in the Rio Grande do Sul, Brazil, 2010 to 2019

Risk factors	Cases		Deaths	*p*-value^1^	Relative risk (95% CI)
	n	%			
Contact with mud	1,574	33.1	62	0.246	-
Animal husbandry	2,161	45.4	80	0.070	-
Water tank	365	7.7	15	0.519	-
Pit, grease trap or sewer	598	12.6	39	0.003	0.573 (0.399 – 0.824)
Location with signs of rodents	3,209	67.4	127	0.119	-
Planting/harvesting	1,724	36.2	57	0.017	1.420 (1.034 – 1.951)
River, stream, pond or dam	1,848	38.8	43	0.000	2.415 (1.709 – 3.413)
Direct contact with rodents	1,339	30.6	69	0.019	0.711 (0.523 – 0.966)
Grain/Food Storage	1,273	26.7	42	0.050	1.355 (0.954 – 1.923)
Wasteland	1,262	26.5	50	0.440	-
Garbage/Rubble	1,225	25.7	67	0.005	0.655 (0.482 – 0.889)
Infected humans	299	6.3	9	0.175	-
Infected animals	48	1.0	1	0.400	-

^1^chi-squaretest, *CI* confidence interval

**Fig. 3:**
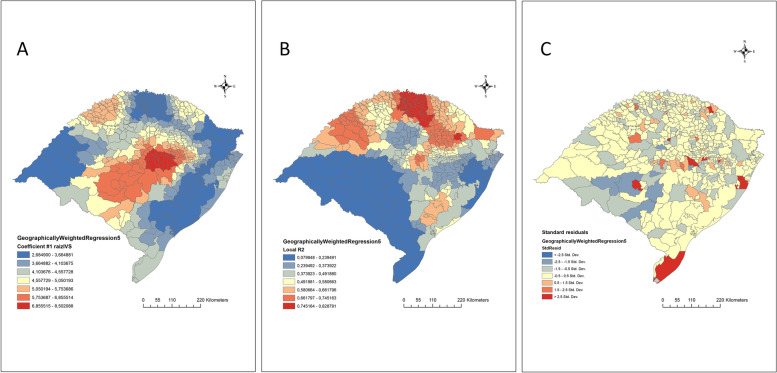
Geographically weighted regression maps of health vulnerability index (HVI) (independent variable) and leptospirosis incidence rate (dependent variable) between municipalities in Rio Grande do Sul, Brazil. **A** β1 coefficient; **B** R2 location; **C** standard residuals (Source; Shape file from IBGE, 2019, done using Qgis version 3.28). https://www.ibge.gov.br/geociencias/downloads-geociencias.html

After applying the OLS regression, it was found that the HVI was positively associated with the incidence rate of leptospirosis in the Rio Grande do Sul (*p* = 0.0001; β_0 =_ 0.085; β_1 =_ 4.6; R^2 =^ 0.36). Figure 3 presents the results of the GWR regression, the maps of the distribution of this relationship according to R^2^, β_1,_ and error parameters in different municipalities. It was observed that the HVI was more representative (R^2^) of the incidence rate in municipalities located in the northern region, mainly in the Northwest and Northeast Rio Grandense mesoregions, while the strength of association (coefficient β1) was greater in municipalities located in the center of the state, mainly in the Centro-oriental Rio Grandense mesoregion.

## Discussion

The present study has provided an overall perception of the determinants of leptospirosis in Rio Grande do Sul, Brazil, between 2010 and 2019. Our results suggested that although the occurrence of leptospirosis in the state of Rio Grande do Sul is significantly associated with various host characteristics, such as male gender and working-age group, the social and environmental determinants remain the prime variables like low education level and ineffective handling of rodents, sewage, and garbage [23–27]. It is evident that this disease mainly affects the people with the aforementioned profile, has been occurring with a high incidence and lethality rate in the Rio Grande do Sul over the last few years. Recent evidence indicates that people who develop leptospirosis usually live in areas with inadequate sanitation and poor housing conditions and are at a high risk of exposure to rodents, contaminated soil, and water [6, 28]. Thus, our study highlights the importance of assessing the effectiveness of various health indicators, such as the HVI, used in public health surveillance systems in promoting primary population-based interventions for this disease.

In the present study, the disease occurrence was higher in white skin color individuals, whereas the relative lethality risk was greater in black skin color people. According to a study by Batista [29], the quality of life, as well as inadequate social conditions like poor living conditions, can have a major impact on the health of a population, thereby increasing the mortality risk. Leptospirosis is a disease directly related to social determinants of health (overcrowding, violence, low education level, and poverty) [6]. In Brazil, the socioeconomic factors determine the lifestyle of the population and serve as a precise measure of poverty and vulnerability that influence health outcomes. Likewise, economic determinants can significantly affect individuals’ exposure to environmental risk factors that directly or indirectly affect their health [30, 31].

Literacy is a critical variable that helps to access and understand health education programs and improve socioeconomic conditions, leading to better efficiency in disease prevention and implementation of disease control campaigns [3]. Furthermore, our results reported that people with incomplete elementary school education represented the largest share of human leptospirosis cases, while people with a lower level of literacy showed a higher mortality risk. Gutiérrez et al. [32] observed a negative relationship between leptospirosis occurrence and educational qualifications; educational coverage and performance in standardized teaching exams were related to a lower incidence of human leptospirosis [32, 33]. Additionally, in Brazil, the increase in school attendance, low school dropout rates, and age-grade distortion in primary education are associated with access to basic sanitation and health care services. Children who survive the disastrous consequences of environmental hazards conducive to the spread of waterborne diseases become ill in conditions that are reflected in epidemiological indicators [34].

In southern Brazil, leptospirosis incidence in rural areas is twice as high as that in urban areas [29]. Approximately 50% of the municipalities in Rio Grande do Sul are considered at risk for the disease, most of which are critical areas for leptospirosis [12]. In the present study, it was observed that most cases occur in rural areas [30, 35], either through direct contact with infected livestock or indirectly through food or the environment. Even though efforts have been made for worker health in Brazil, the control of diseases acquired at work is a challenge in most Brazilian states [36, 37]. Specifically in relation to infectious diseases, such as leptospirosis, the risk of transmission is also related to the non-use of personal protective equipment [38, 39]. In addition, there is also low inspection in these areas, and measures that encourage the correct use of equipment, considering that its use can avoid direct interaction with pathogenic agents, such as *Leptospira sp.* [40], and decrease the risk of professionals acquiring occupational diseases. Therefore, preventive strategies to guarantee a safe working condition, such as provision and supervisory actions to ensure compliance with usage, must be incorporated and intensified together with the promotion of educational campaigns.

Epidemiological data show a change in the location of infection over the years, being acquired more in the home environment than at workplaces [24]. Similar to the findings of the present study, Magalhães & Acosta [26] observed that in Porto Alegre (RS), approximately half of the leptospirosis cases occurred in the house environment, followed by the cases that were linked to the workplace. Identification of the factors that participate in the dynamics of *Leptospira* transmission is extremely important, as it can contribute to improving the knowledge of decision-makers in healthcare services in controlling this disease. The risk factors that stood out in this study mainly concern exposure to areas without sanitation and places of possible contact with rodent urine. It is also highlighted that the rapid urbanization and unplanned population growth, now being observed in most Brazilian municipalities, leads to the emergence of poor housing conditions [41] in disease-prone areas that are subjected to risk factors for leptospirosis and other infectious diseases [42]. Another recurrent negative impact due to increasing urbanization is the onset of floods, which cause serious environmental damage by favoring the growth and migration of vectors of diseases and epidemics [9], enhanced by poor hygiene and sanitation services; thus, exposing the entire population to serious health risks [43, 44].

Health vulnerability is described as the process of being at risk for illness or changes in physical and mental health conditions as a result of economic and social determinants [45]. To plan public health policies, some municipalities classify their population in terms of vulnerability using the HVI, which associates population indicators with health indicators [46]. It was found that the HVI is associated with a higher incidence rate of leptospirosis, especially in municipalities located in the Nortwest, Northeast andCentro-oriental Rio grandense mesoregion. To our knowledge, there are no studies that have assessed the relationship between social vulnerability measured by the HVI and the incidence of leptospirosis in the state to date. Therefore, the health vulnerability index can be used as a tool to help municipalities identify priority areas for intervention and resource allocation and develop effective population-based prevention strategies for positive health outcomes [47]. In addition, these results reinforce that the control of leptospirosis depends, mainly, on the reduction of vulnerability and social inequality in the country, through improvements in quality of life, access to infrastructure, such as housing, sanitation in urban and rural areas, education, and income [48, 49] in addition to measures directly related to secondary prevention, such as early diagnosis and universal treatment for the population.

A high rate of the disease has already been described in the central region, which produces tobacco and contains a hydrographic basin with tributaries recognized by periodic flooding, so it was suggested that leptospirosis should also be considered in natural disasters management strategies for municipalities in this region [30]. Furthermore, a high incidence was also observed in the mesoregions located in the northeast and northwest of the state, which are characterized by having an economy focused on agriculture and livestock [50, 51] with large and medium-sized properties that cultivate soybeans, wheat and tobacco, and swine. These characteristics favor the establishment of *Leptospira* in the environment, as reported in the study by Mesquita et al. [52], who evaluated the morbidity of leptospirosis in cattle, and demonstrated a high prevalence in the northwest mesoregions, followed by the northeast and southeast, with a consequent risk to workers involved in these productive activities in these regions [53].

Another study by Buffon [3] assessed the socio-environmental vulnerability to leptospirosis and identified areas at risk of flooding, thus, relating the disease burden with determinants of the disease. In the Rio Grande do Sul, Barcellos et al. [35] evaluated the spatial distribution of leptospirosis with geographic characteristics, observed a higher number of cases in municipalities located in the center and south of the state, and suggested the ecological characteristics favorable to the transmission of the disease are the areas prone to the proliferation of synanthropic rodents and intensive agricultural production [54, 55].

Using secondary data from surveillance systems implies working with numerous limitations, mainly related to underreporting bias and incomplete recording of information [6, 7]. Thus, the municipal epidemiological surveillance systems and the primary health services must be efficient and sensitive enough to successfully manage disease outbreaks [36, 56] The professionals should be correctly trained to identify and investigate suspected cases of the disease to ensure early diagnosis and timely treatment [56]. Additionally, occasional surveillance and epidemiological investigations along with health education and disease prevention programs should be conducted for the entire population [36]. Despite this, in Brazil, most surveillance programs for neglected diseases in developing countries still suffer from lack of funding and other challenges, like migration, poorly planned urbanization, social inequality, poor access to basic sanitation, climate changes and deforestation, with impacts on control actions [53, 55, 57–59].

To our knowledge, no previous study to date has examined the association between leptospirosis incidence and HVI in the Rio Grande do Sul, Brazil. The lack of knowledge about the impact of leptospirosis in some municipalities reduces the importance of socioeconomic determinants, resulting in less effective measures to control the disease. Accurate risk assessment is extremely crucial for the development of effective population-based prevention strategies, and specific control policies for the most vulnerable populations [9, 48]. The epidemiological surveillance services should also guide the disease-prone individuals in the workplace settings to adopt control measures like using adequate protective equipment for better health outcomes.

Our study have examined the association between leptospirosis incidence in southern Brazil with various environmental and socioeconomic factors. Through the use of spatial regressions models, we intend to help the understanding of disease occurrence in an endemic area in southern Brazil. Future studies could use the results obtained through these models to evaluate smaller geographic areas, particularly those in which the relationship between social vulnerability measured by HIV and leptospirosis is stronger, as in Northwest, Notheast and Centro-oriental Rio Grandense mesoregions. Some of the limitations of this study were the difficulty in providing accurate information about all the variables in the database, as well as the lack of uniformity in data collection and subsequent database entries. However, even with the slightest notification biases, the analysis of study data might be extremely valuable for health agencies to analyze the behavior of various health problems and direct efforts and resources for more effective surveillance and control measures, in addition to anticipating risk situations on time.

## Conclusion

The incidence of leptospirosis is significantly associated with characteristics of the host, such as male sex, productive age group, low level of education and with environmental determinants such as living in rural areas and relationship with work. In addition, lethality from the disease was higher in elderly people, with black skin color and with low education, and in individuals with activities related to agriculture and food storage and in contact with flooded areas.

Furthermore, in Rio Grande do Sul leptospirosis is significantly related to the municipal HVI, mainly in the Northwest, Northeast and Centro–oriental Rio Grandense mesoregions.

## Data Availability

All data generated or analyzed during this study are available within the article and its supplementary information files.

## Abbreviations

SINAN: Notifiable Diseases Information System
RS: Rio Grande do Sul
HVI: Health vulnerability index

## Footnotes

1 Sistema de Informacao de Agravos de Notificacao (SINAN), in Portuguese.
